# VTA network dominance in depression confers distinct psychopathological states through blunted neural tracking of reward prediction errors

**DOI:** 10.21203/rs.3.rs-7166201/v1

**Published:** 2025-09-09

**Authors:** J. Beltrán, M. Li, Y. Jacob, G. Butler, M. Mehta, J.W. Murrough, A. Radulescu, L.S. Morris

**Affiliations:** 1. Department of Psychiatry, Icahn School of Medicine at Mount Sinai, New York, NY, USA; 2. Nash Family Department of Neuroscience & Friedman Brain Institute, Icahn School of Medicine at Mount Sinai, New York, NY, USA; 3. BioMedical Engineering and Imaging Institute, Department of Radiology, Icahn School of Medicine at Mount Sinai, New York, NY, USA; 4. Center for Computational Psychiatry, Icahn School of Medicine at Mount Sinai, New York, NY, USA; 5. The Laureate Institute for Brain Research, Tulsa, OK, USA; 6. VISN 2 Mental Illness Research, Education and Clinical Center (MIRECC), James J. Peters VA Medical Center, Bronx, NY, USA; 7. Nuffield Department of Clinical Neurosciences, University of Oxford, UK; 8. Department of Experimental Psychology, University of Oxford, UK

## Abstract

Decades of preclinical research have implicated divergent roles of basal and task-based activity within the mesolimbic and mesocortical dopamine pathways in the neurobiological mechanisms of depression. However, translation to humans has been limited by low-resolution neuroimaging methods during the inherent computational nature of learning processes. Here, utilizing high-resolution, precision multi-echo functional MRI, Bayesian modeling, and graph-theory network influence modeling, we demonstrate that relative to controls, individuals with major depressive disorder (MDD) are characterized by an elevated influence of the ventral tegmental area (VTA) within the mesolimbic/mesocortical network during task-free brain states. During task-based brain states, mesocortical hyperconnectivity was linked to blunted neural tracking of reward prediction error (RPE) signals in the nucleus accumbens and greater anhedonia, while blunted neural tracking of RPEs in the basolateral amygdala was linked with greater anxiety. Thus, mesocortical hyperconnectivity in MDD may lead to a computationally underutilized learning mechanism that impacts discrete depression and anxiety symptom domains. Together these results may explain paradoxical preclinical work and elucidate neural underpinnings for the clinical heterogeneity of depression.

Decades of research in animals have demonstrated that the dopamine (DA) system is critical for mediating motivation, reward processing, and behavioral phenotypes underlying anhedonia^1–9^. The DA system stems largely from the ventral tegmental area (VTA) and substantia nigra (SN), two small structures deep in the midbrain^10^. DA projections from the VTA to limbic regions including the nucleus accumbens (NAc), amygdala and hippocampus form the mesolimbic pathway^2,3^ (Figure 1A). While dopamine projections to the prefrontal cortex, including the ventromedial and dorsomedial prefrontal cortex (vmPFC/dmPFC), prelimbic and subgenual anterior cingulate cortex (plACC/sgACC), form the mesocortical pathway (Figure 1A). Together these pathways modulate a variety of distinct reward-related and aversion-related motivational processes^11,12^.

In preclinical models of depression using the chronic social defeat stress (CSDS) model, mice that develop a depressive-like phenotype, characterized by reduced expression of social interaction and sucrose preference^13–19^, demonstrate increased basal firing rates of VTA-dopamine (DA) neurons within the mesolimbic pathway targeting the NAc^16,18^. On the other hand, mice that develop an anxiety-like phenotype, characterized by altered approach/avoidance balance solely or in addition to depressive-like behaviors^16,20^, demonstrate decreased VTA firing to the basolateral amygdala (BLA). Within the mesocortical pathway, aversive stimuli such as stress increase extracellular DA in the mPFC of mice that underwent CSDS^21^, and increase synaptic strengthening between the VTA and mPFC in response to aversion^22^, suggesting increased signaling and connectivity between these regions. While VTA-DA firing in response to unexpected rewards is a well-established mechanism underlying adaptive learning^23^, excessive firing/hyperconnectivity of these same neurons has been postulated to paradoxically reflect a reduced capacity for reward learning in conditions characterized by dopaminergic dysfunction, such as alcohol use disorder^24^. As such, impaired reward learning may stem from a limited dynamic range in VTA signaling, limiting the diversity of firing patterns required to confer reward-related information to target regions^24^. While this has not been empirically demonstrated in preclinical or human studies, this body of preclinical work suggests that distinct VTA pathway alterations might produce specific behavioral and symptomatic phenotypes, explaining in part the heterogeneity observed in individuals with MDD^20,25^.

MDD is a highly prevalent disorder in humans, with marked sex differences^26,27^ and accumulating evidence linking sex hormonal levels to severity and specific phenotypes^28–30^. However, understanding the neurobiological mechanisms underlying MDD and these sex-specific patterns remains limited in part due to technical challenges in mapping brain function with sufficient spatial and temporal resolution. Recent developments in precision functional mapping^31^ have demonstrated the utility of multi-echo (ME) based acquisition and analysis for precise delineations of neural networks in humans with depression. The integration of ME-based acquisition and denoising efforts with high-field 7T fMRI capitalizes on the enhanced signal-to-noise ratio, spatial resolution, and enhanced T2* contrast that can be obtained, while addressing limitations of conventional 3T fMRI and 7T-specific artifacts by sampling shorter echo times, reducing slice thickness, and utilizing robust denoising based on physical principles. Within the midbrain, 7T exhibits shorter T2* values than 3T (11–14ms versus 20–25ms), highlighting the benefits of ME and 7Ts ability to amplify differences between task-free and task-based brain states while maintaining whole-brain coverage and ensuring optimal T2* sampling across the whole brain. Recent findings further demonstrate superior test-retest reliability of shorter-duration ME acquisitions compared to single-echo protocols, particularly in clinically relevant regions^31^. This is attributable to ME’s capacity to minimize thermal noise, regional T2* decay variability, and S0-related artifacts.

Here we developed a high-resolution multi-echo whole-brain functional mapping protocol with specificity and power for imaging discrete VTA circuits (covering midbrain, subcortex and cortex). We then applied computational neurobehavioral modeling and precise deep-phenotyping to parse how specific symptoms map onto the functional roles of mesolimbic/mesocortical activity during task-free and task-based brain states. Using graph theory models, we first demonstrate a broad network of hyperconnectivity within the VTA-centered mesocorticolimbic network in depression, and an elevation in influence of VTA on the network, which is specifically associated with the anhedonia phenotype and mirrors patterns observed in preclinical models of depression. Next, we demonstrate that mesocortical hyperconnectivity is specifically associated with reduced neural tracking of reward prediction errors (RPEs) in the NAc. This finding suggests that mesocortical hyperconnectivity may impair the utility of NAc-derived RPEs for guiding learning and adaptive behavior, providing evidence for a neural mechanism that limits the dynamic range required for effective information relay. Then, as predicted from preclinical work, we link aberrant neural tracking of RPEs in the NAc and BLA in the human brain to specific phenotypic domains of anhedonia and anxiety, respectively. Finally, we demonstrate that elevated levels of estradiol are associated with VTA hyperconnectivity and VTA dominance during task-free brain states, physiologically explaining some long-known sex differences in depression pathophysiology. Together, this highly translational work bridges the gap from preclinical to clinical findings and explains some notable paradoxical findings whereby mesocortical hyperconnectivity is associated with an underutilized neurocomputational mechanism that has been consistently linked with behavioral deficits in sensitivity to reward and reward learning. These results also point to specific neural pathways that underlie distinct phenotypes and contribute to efforts to parse symptom heterogeneity in depression.

## VTA Network Dominance in Depression

**Figure 1.**
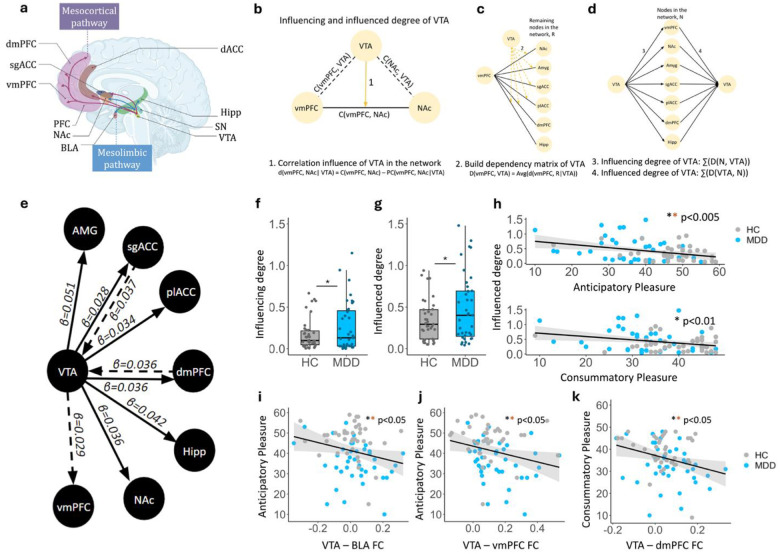
VTA network dominance in MDD. **a** Schematic of mesocortical and mesolimbic pathways. **b-d** the DEPNA method. **e** DEPNA results illustrating VTA influence on the network is greater in MDD than HC. Dashed lines indicate trends at p < 0.07. **f-h** VTA influencing and influenced degree is significantly greater in MDD than HC, with the influenced degree showing negative relationships with anticipatory and consummatory pleasure. **i-k** VTA-BLA and VTA-vmPFC FC are negatively associated with anticipatory pleasure meanwhile VTA-dmPFC FC is negatively associated with consummatory pleasure. ** *survived FDR correction*. C = correlation, PC = partial correlation, HC = healthy control, MDD = major depressive disorder, dmPFC = dorsomedial prefrontal cortex, sgACC = subgenual anterior cingulate cortex, vmPFC = ventromedial prefrontal cortex, PFC = prefrontal cortex, NAc = nucleus accumbens, BLA = basolateral aymgdala, dACC = dorsal ACC, Hipp = hippocampus, SN = substantia nigra, VTA = ventral tegmental area

We applied the Dependency Network Analysis (${\text{D}}_{\text{EP}}\text{NA}$) (Figure 1B–D) to high-resolution task-free (i.e. resting state) fMRI time series data to assess directional dependencies between the VTA and regions within the mesocorticolimbic network in MDD compared to healthy controls (HC). Our results demonstrated significantly higher influencing and influenced degrees of the VTA on a broad network of target regions along both the mesolimbic and mesocortical pathways in MDD compared to HC (Figure 1E–G, $\beta_{\text{influencing}}=0.153,p_{\text{influencing}}=0.028;\beta_{\text{influenced}}=0.201,p_{\text{influenced}}=0.040$). Traditional functional connectivity estimates also revealed a consistent pattern of hyperconnectivity during task-free brain states within both pathways in MDD at the whole-brain level (Supplementary Figure 1) and at the ROI-level within the ventral mesocortical pathway before FDR correction (Supplementary Table 2 and 3, $F_{\text{sgACC}}=4.590,p_{\text{uncorr}}=0.036,p_{\text{FDR}}=0.178;\beta_{\text{vmPFC}}=0.073,p_{\text{uncorr}}=0.044,p_{\text{FDR}}=0.190$). Together, these findings reflect a critical increased shift in influence towards dominance of VTA on the target network in MDD, similar to that observed in preclinical models of depression.

This broad network of VTA hyper-dominance was further associated with anticipatory and consummatory anhedonia in distinct ways. First, for the network as a whole, the ${\text{D}}_{\text{EP}}\text{NA}$ revealed that both anticipatory and consummatory anhedonia were significantly associated with a higher influenced degree of the VTA on the network (Figure 1H, Supplementary Figure 2, $\beta_{\text{anticipatory}}=-0.013,p_{\text{anticipatory}}=0.001,p_{\text{FDR}}=0.002;\beta_{\text{consummatory}}=-0.013,p_{\text{consummatory}}=0.006,p_{\text{FDR}}=0.006$), and not with its influencing degree (see Supplementary Table 4 for full results and Supplementary Figure 2 for additional confirmatory model results). Second, there was a significant relationship between higher anticipatory anhedonia (TEPS-A) and increased connectivity with a range of mesocortical and mesolimbic targets including the BLA ($r=-0.271,p_{\text{uncorr}}=0.019,p_{\text{FDR}}=0.044$), plACC ($r=-0.241,p_{\text{uncorr}}=0.038,p_{\text{FDR}}=0.045$), sgACC ($r=-0.247,p_{\text{uncorr}}=0.034,p_{\text{FDR}}=0.045$), hippocampus ($r=-0.327,p_{\text{uncorr}}=0.004,p_{\text{FDR}}=0.015$), dmPFC ($r=-0.333,p_{\text{uncorr}}=0.004,p_{\text{FDR}}=0.015$), and vmPFC ($r=-0.261,p_{\text{uncorr}}=0.025,p_{\text{FDR}}=0.044$) (Figure 1I–J, see Supplementary Figure 3 and Supplementary Table 5 for full results). Meanwhile, consummatory anhedonia (TEPS-C) was specifically associated with dorsal mesocortical hyperconnectivity (Figure 1K, $r_{\text{dmPFC}}=-0.311,p_{\text{uncorr}}=0.007,p_{\text{FDR}}=0.048$). A similar pattern of findings was observed in a confirmatory follow-up model (Supplementary Table 6). Contrary to expectation, there was no significant correlation between VTA circuitry connectivity and overall depressive (MADRS) or anxiety (STICSA) symptoms (Supplementary Table 7). Thus, these findings indicate a selective specificity of the VTA-centered network for anhedonia, consistent with preclinical and clinical evidence of dopaminergic dysfunction underlying reward-related and motivation deficits across various psychiatric disorders (e.g., depression, schizophrenia and addiction)^32–35^.

## Specific behavioral/computational reward learning deficit

Next, bi-valent trial and error learning was assessed via a two-armed probabilistic bandit task with intermixed trials for reward, loss and neutral outcomes (Supplementary Figure 4), combined with the same high-field multi-echo whole-brain functional MRI protocol. During task-based fMRI, participants learned to maximize payoffs in an 80/20% probabilistic choice context within a dynamic task environment of intermixed reward, loss and neutral outcomes.

**Figure 2.**
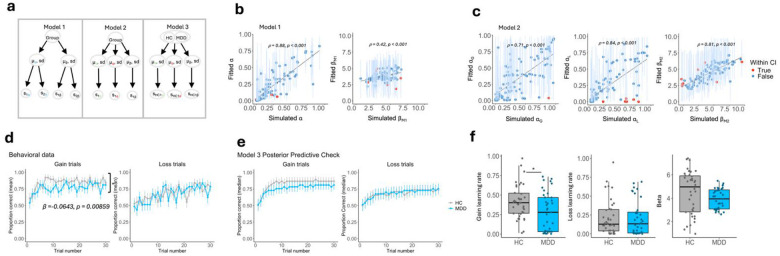
Reward learning deficit in MDD. **a** Hierarchical Bayesian framework of the three computational models tested. **b-c** Parameter recovery for competing models 1 and 2. Blue data points indicate values that fall within the 95% credible intervals (CI) of the fitted parameters, while red datapoints indicated values outside the 95% CI. **d** Trial by trial learning curves across gain and loss trials whereby MDD participants make fewer correct choices during gain but not loss trials. **e** Model 3 posterior predictive check. **f** Subject-level learning rate and beta parameter estimates generated using model 3, whereby MDD participants demonstrate reduced learning rates during gain trials, compared to HC. *HC = healthy control, MDD = major depressive disorder*

Three computational Q-learning models^36^ were formulated and fitted to participant’s behavioral data within a hierarchical Bayesian framework, using Hamiltonian Monte Carlo estimation via RStan^37^ (Figure 2A, see Supplementary Methods). Model parameters were derived to capture trial-by-trial differences between expected and actual outcomes (RPEs), individual learning rates (α) and outcome sensitivities (beta, β). Parameter recovery analysis demonstrated that all parameters were independently estimated and could successfully be recovered (**Figure B-C**). The winning model was determined using the Widely Applicable Information Criterion (WAIC)^38,39^ and a posterior predictive check which demonstrated that a model with two learning rates dependent on stimulus trial type and one outcome sensitivity parameter effectively captured choice accuracy performance across reward and loss trials, as expected from the empirical data (Figure 2D–E). For further details on the computational modeling see Supplementary Methods and Supplementary Figure 5.

A specific deficit in positive-valence learning (reward-learning) but not negative-valence learning (loss-learning) was observed in MDD compared to controls (Linear mixed effects model, group x trial type interaction: $\beta =-6.43\times 10^{-2},\text{t}=-2.63,\text{p}=0.008$) (Figure 2D). Computational estimates of positive-valence learning rates were reduced in the MDD group in comparison to controls (Figure 2F, W=910, p=0.0281), with no group difference in loss-learning (Figure 2F, W=723, p=0.837) or outcome sensitivity (Figure 2F, W=853, p=0.114).

**Figure 3.**
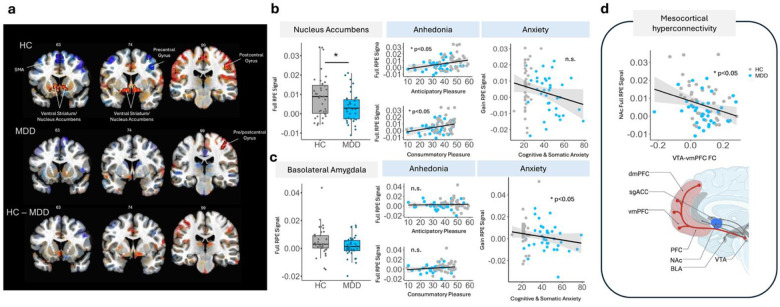
Symptom specificity of neural computations in depression. **a** Whole brain analysis whereby HC (but not MDD) demonstrate RPEs encoded in the NAc (Left peak: xyz=−13.5 −0.5 −15.5, Z=4.07; Right peak: xyz=+16.5 −0.5 −15.0, Z=4.24; with voxelwise p<0.001). Images are displayed with translucent statistical thresholding whereby clusters outlined in black are statistically significant (voxelwise p < 0.001, alpha < 0.05) meanwhile sub-threshold clusters are illustrated with decreasing opacity as statistical significance decreases. Values displayed above brain images indicate slice number. **b** MDD participants exhibit reduced neural tracking of RPEs in the NAc which are associated with worse symptoms of anhedonia, but not anxiety. **c** Reduced neural tracking of RPEs during gain trials in the BLA are associated with worse anxiety, but not anhedonia. **d** Increased VTA-vmPFC FC during task-based brain states is associated with decreased neural tracking of RPEs in the NAc. *HC = healthy control, MDD = major depressive disorder, RPE = reward prediction error*

In a confirmatory analysis of a reward-learning impairment in MDD, model-based fMRI utilizing the trial-by trial RPEs across the full task (i.e. ‘Full RPE’) as parametric regressors was conducted. Whole-brain corrected voxelwise analyses demonstrated significant encoding of RPEs in the NAc and right postcentral gyrus in HC but not MDD (Figure 3A, Left peak: xyz=−13.5 −0.5 −15.5, Z=4.07; Right peak: xyz=+16.5 −0.5 −15.0,Z=4.24, with voxelwise p<0.001, see Supplementary Table 8 and Supplementary Figure 6 for additional results). Meanwhile, targeted ROI analyses suggested that participants with MDD demonstrated reduced neural tracking of RPEs in the NAc specifically (W = 882, p = 0.033), in line with previous work^40,41^ (Figure 3B, Supplementary Table 9).

Together these results coincide with accumulating evidence that MDD is associated with aberrant reward learning specifically, and questions prevailing theories about loss sensitivity/learning in MDD.

## Mesocortical hyperconnectivity links with blunted neural tracking of RPEs: limited dynamic range

Next, high-field precision multi-echo, model-based fMRI utilizing the trial-by trial RPEs across the full task (i.e. ‘Full RPE’) as parametric regressors was applied to assess how task-based mesocortical hyperconnectivity and VTA network dominance affect bi-valent learning processes. This analysis revealed an association between increased mesocortical connectivity and decreased neural tracking of RPEs in the NAc (R=−0.29, p=0.011, ${\text{p}}_{\text{FDR}}=0.044$) (Figure 3D, Supplementary Table 10). Further, while mesolimbic connectivity (NAc and BLA) was positively associated across task-free and task-based brain states (${\text{rho}}_{\text{VTA-NAc}}=0.285,\text{p}=0.0178;{\text{rho}}_{\text{VTA-BLA}}=0.239,\text{p}=0.048$), ventral mesocortical connectivity (VTA-vmPFC) became dissociated between brain states (Supplementary Figure 7, ${\text{rho}}_{\text{VTA-vmPFC}}=0.129,\text{p}=0.290$).

Together, these findings suggest a functional decoupling of mesocortical, but not mesolimbic, connectivity across brain states, and points to mesocortical hyperconnectivity as an underlying neural mechanism that may limit the dynamic range through which reward learning signals are transmitted across the brain.

## Symptom specificity of neural computations in depression

Preclinical studies using CSDS models demonstrate that increased neuronal firing from the VTA to the NAc promotes behavioral features of anhedonia, such as reduced sucrose preference and social interaction^18^. Meanwhile, decreased neuronal firing from the VTA to the BLA promotes anxiety but not depressive-like phenotypes, as evidenced by decreased time spent in the open arms of an elevated plus maze with no change in depressive-like behaviors^18,20^. To determine whether these circuit-specific findings translate from animals to humans, we conducted analyses estimating associations between RPE encoding within NAc/BLA and depression/anxiety symptoms. Blunted neural tracking of RPEs in the NAc, but not BLA, was associated with anticipatory and consummatory anhedonia (Figure 3B, TEPS-A: rho=0.260, p=0.0253, ${\text{p}}_{\text{FDR}}=0.0274$; TEPS-C: rho=0.257, p=0.0274, ${\text{p}}_{\text{FDR}}=0.0274$, see Supplementary Table 11–12 for within-group-level associations). Meanwhile, blunted neural tracking of reward-learning RPEs in the BLA, but not NAc, was associated with worse anxiety (Figure 3C, Supplementary Table 13, rho=−0.245, p=0.0352). Taken together, these findings demonstrate a cross-species dissociation of neural circuits which are associated with distinct symptoms of depression and anxiety. Additional correlations between gain and loss RPEs and anhedonia were tested (see Supplementary Figure 8).

## Effects of sex hormones

**Figure 4.**
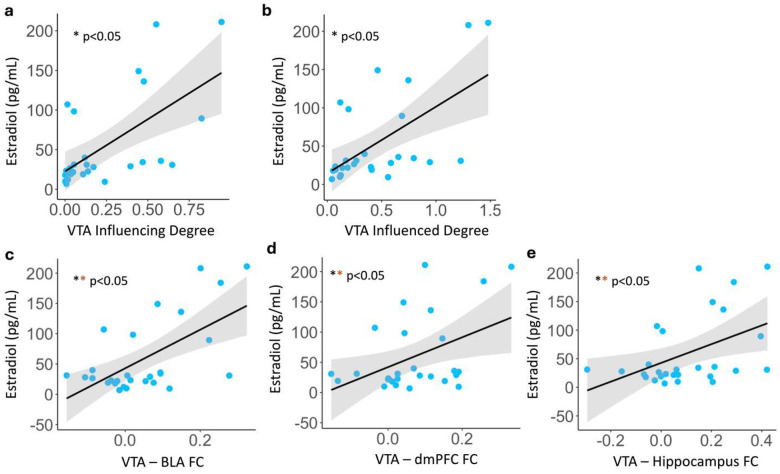
Sex hormones and MDD. **a-b** VTA influencing and influenced degree is positively associated with increased estradiol levels in MDD (N=27). **c-e** Functional connectivity of VTA-BLA, VTA-dmPFC and VTA-hippocampus during task-free brain states are positively associated with increased estradiol levels in MDD (N=28). ** *survived FDR correction*

To address the underlying biological differences between sexes, endogenous gonadal hormone levels in the periphery were derived and estimated on the morning of the high-resolution scan. Circulating estradiol levels exhibited significant associations with both the influencing and influenced degrees of the VTA within the MDD group (Figure 4A–B, $r_{\text{influencing}}=0.526,p_{\text{influencing}}=0.008;r_{\text{influenced}}=0.556,p_{\text{influenced}}=0.005$) and across all participants (Supplementary Figure 9, $r_{\text{influencing}}=0.317,p_{\text{influencing}}=0.034;r_{\text{influenced}}=0.380,p_{\text{influenced}}=0.011$). Separate analyses across female and male participants demonstrated similar notable relationships between estradiol and VTA influence within each sex (Supplementary Table 15).

Moreover, circulating estradiol levels were positively associated with mesocortical hyperconnectivity within the ventral mesocortical pathway (VTA-vmPFC) across all participants (Supplementary Figure 9, Supplementary Table 14, $r=0.388,p_{\text{uncorr}}=0.008,p_{\text{FDR}}=0.054$). In the MDD group, estradiol was highly correlated with increased dorsal mesocortical and mesolimbic functional connectivity, including the VTA and BLA (Figure 4C, $r=0.650,p_{\text{uncorr}}=0.000,p_{\text{FDR}}=0.003$), dmPFC (Figure 4D, $r=0.488,p_{\text{uncorr}}=0.013,p_{\text{FDR}}=0.031$), and hippocampus (Figure 4E, $r=0.521,p_{\text{uncorr}}=0.008,p_{\text{FDR}}=0.027$). Associations between estradiol levels and VTA circuitry connectivity were also examined separately by sex, which revealed a positive correlation between higher estradiol levels and VTA-to-BLA hyperconnectivity in female MDD patients (Supplementary Table 14, $r=0.682,p_{\text{uncorr}}=0.007,p_{\text{FDR}}=0.050$). We further explored the association between estradiol levels and VTA circuitry connectivity respectively by sex, and found that higher estradiol levels were positively correlated with hyperconnectivity between VTA and BLA in female MDD patients (Supplementary Table 14, $r=0.682,p_{\text{uncorr}}=0.007,p_{\text{FDR}}=0.050$). While there were no associations between estradiol and two subtypes of anhedonia, nor any significant findings of circulating testosterone (Supplementary Table 16–18), these results suggest that fluctuations in estradiol levels may be a crucial mechanism for modulating the increased functional connectivity and VTA influence in MDD.

## Discussion

Mounting evidence from preclinical models of depression characterizes depressive-like behaviors by dysregulated mesocortical and mesolimbic circuitry and implicates distinct VTA subcircuits in the presentation of anhedonic and anxiety-like phenotypes^16,18,20,22,42–44^. The modular composition of the VTA can explain this circuit-based dissection of specific depressive phenotypes^45^ and is critical to the identification of treatments that can selectively target specific symptoms. However, investigating whether VTA subcircuits play a similar role in human populations has remained challenging, in part due to the VTA’s lack of precise anatomical boundaries^46^ and poor imaging resolutions. Herein, using ultra-high field (7T) MRI to image small brain structures with high precision in humans, we present the first translational and replicative evidence of preclinical work demonstrating VTA network alterations in depression. We then extend upon these findings by linking mesocortical hyperconnectivity during task-based brain states to an underutilized neurocomputational mechanism that disrupts bi-valent learning processes across the NAc and BLA to promote anhedonia and anxiety symptoms. Together, these results draw parallels between preclinical and clinical works and strengthen the translational utility of high-field neuroimaging for elucidating MDD pathophysiology and identifying treatment options for individuals.

A substantial proportion of individuals with MDD fail to respond to standard first-line antidepressants. This can be due to various factors, including comorbid mental and physical disorders, which are highly prevalent in MDD^47^. Indeed, individuals with comorbid anxiety disorders present with more severe depression and are less likely to respond to treatment^48^. These observations underscore the need for biologically informed treatment approaches that move beyond treating categorical diagnoses and instead target neural processes that mediate symptom presentation/behavior. Herein, we demonstrate that mesocortical hyperconnectivity during task-based brain states is linked with a disturbed balance of learning efficiently from rewards and losses. These findings align with preclinical evidence showing that mesocortical dopamine mediates distinct facets of decision-making such as behavioral flexibility and reinforcement of rewarded actions, via distinct dopaminergic receptors and projections to subcortical projection targets including the NAc and BLA^49^. Bi-valent learning is a critical facet of adaptive decision-making in dynamic environments. However, this process may be underutilized in depression and anxiety phenotypes, leading to behavioral impairments. In anxiety disorders, this can be characterized as avoidance learning in an effort to minimize losses^50^. Meanwhile, in depressive disorders, this can be characterized as attentional biases towards losses or reduced sensitivity for rewards^35,51–56^. Together, these biases may reflect a behavioral phenotype that stems from increased mesocortical connectivity but results in distinct phenotypes depending on the dopaminergic target that is modulated.

Our results suggest different types of therapeutic interventions may be beneficial for regulating network nodes related to future reward prediction versus immediate reward experience. In individuals that present with anticipatory anhedonia or anxiety, regulating network nodes related to future reward (e.g., vmPFC, BLA and hippocampus) may be beneficial. Meanwhile, in individuals who present with consummatory anhedonia, greater consideration for regulating regions involved in immediate reward experience (e.g., dmPFC and NAc) may help enhance the motivational drive to seek subsequent rewards. Accumulating evidence from pharmacological studies investigating the effects of a KCNQ-selective channel opener, ezogabine, demonstrates its therapeutic potential for alleviating symptoms of depression and anticipatory anhedonia in particular. This is achieved by reducing the functional connectivity between striatal reward regions and clusters in the midcingulate and posterior cingulate cortex, which connects with the vmPFC^57–60^. Therefore, targeting VTA KCNQ channels may improve reward learning, enable a shift away from negative self-referential thinking, and ultimately restore the homeostatic balance of learning efficiently from rewards and losses.

Beyond pharmacological approaches, non-invasive techniques such as transcranial ultrasound stimulation (TUS) are emerging as promising tools for treating psychiatric disorders. TUS can target both cortical and deep brain regions with high spatial specificity^61,62^ making it advantageous relative to transcranial magnetic stimulation (TMS) and transcranial direct current stimulation (tDCS). Indeed, previous studies in healthy participants have demonstrated that modulation of the right inferior frontal gyrus via transcranial focused ultrasound (a type of TUS) can enhance mood states and reduce functional connectivity with left prefrontal and limbic regions^63^. Therefore, future research may consider TUS for non-invasively modulating the VTA dominance of the mesocorticolimbic network we observed in MDD and in turn, regulate mood in individuals with MDD.

In addition to symptom heterogeneity, sex differences may represent a critical factor in tailoring intervention strategies, given the modulatory role of hormonal fluctuations on VTA circuitry^64,65^. In the present study, fluctuations in estradiol were positively correlated with VTA-vmPFC functional connectivity during task-free brain states. Animal research indicates that fluctuations in estradiol levels during the estrous cycle in female mice leads to elevated activity in the VTA, enhances dopamine system activity and amplifies the rewarding effects of cocaine^65^. Therefore, high estradiol levels and their cyclical fluctuations may enhance dopamine system activity, promoting excessive VTA-vmPFC connectivity and amplifying the severity of consummatory anhedonia. This association between hormones and changes in functional connectivity may also explain MDD sexual dimorphism whereby females exhibit higher estradiol levels than males and experience more pronounced depressive symptoms^66^. Furthermore, sex and hormonal variation may play a key role in emotional and reward system regulation. Therefore, interventions targeting estrogen dynamics may also offer insight towards novel treatment strategies for improving symptoms in female individuals with depression.

Taken together, this work bridges the gap between preclinical and clinical works and explains paradoxical findings whereby VTA hyperconnectivity can translate into impaired cognitive processes such as reward learning and anhedonia, which are both hallmark characteristics of MDD. These findings may inform intervention strategies focused on modulating the VTA and its functional connectivity using pharmacological interventions (i.e., ezogabine), hormonal interventions and neuromodulation techniques such as TUS.

## Methods

### Participants

Adult volunteer research participants (ages 18–65) were recruited from the New York City area through the Depression and Anxiety Center for Discovery and Treatment at the Icahn School of Medicine at Mount Sinai (ISMMS). Participants met inclusion criteria if they had no history or current evidence of neurocognitive disorder or substance use disorder, no active general medical problems, no metal in the body or contraindications to magnetic resonance imaging (MRI), and sufficient understanding of the English language. The clinical group consisted of participants who met criteria for a depressive disorder including MDD, persistent depressive disorder or other specified disorder per the Diagnostic and Statistical Manual of Mental Disorders-Fifth Edition (DSM-V) or Mini International Neuropsychiatric Interview (MINI). Participants who were currently experiencing depressive symptoms that are not better explained by another DSM-5 diagnosis but did not meet full criteria for a current depressive disorder were also considered. Healthy control participants had no lifetime history of psychiatric or neurological disorder as defined by the DSM-V or MINI. Additional screening procedures included full medical history, urine pregnancy and menstrual phase (if applicable), and urine drug toxicology. All study procedures were conducted in accordance with the guidelines and regulations set by the Program for Protection of Human Subjects and Institutional Review Board at the ISMMS. Participants provided written informed consent and were compensated for their time.

In total, ninety participants participated in the study. Eighty-three participants successfully completed the full multi-echo 7T resting-state fMRI scan and Reinforcement Learning task (see Table 1). Participants were excluded due to signal quality issues, including low signal-to-noise ratios or signal loss and corruption during preprocessing, therefore, N=78 participants’ resting state data were included for further analyses. Participants who made less than 50% correct choices across both reward and loss trials or failed to make any selection throughout the task were excluded as this may indicate task non-compliance^67^. After applying these exclusion criteria, 7 participants were excluded. Additionally, N=1 was excluded due to obsessive compulsive disorder comorbidity with MDD and N=1 had behavioral data but no imaging data. A total of N=69 (34 HC, 35 MDD) completed both resting state (task-free) and task-based fMRI (see Supplementary Table 19 for details). Sex hormone data for estradiol and testosterone were available for 51 participants (31 females), among which 28 participants had an MDD diagnosis (16 females).

### Clinical assessment

The severity of MDD was assessed using the Montgomery-Åsberg Depression Rating Scale (MADRS)^68^. Anhedonia was measured with the Temporal Experience of Pleasure Scale (TEPS)^69^. Anxiety levels were evaluated using the State-Trait Inventory for Cognitive and Somatic Anxiety (STICSA)^70^.

### Reinforcement Learning task

During the Reinforcement Learning task (Supplementary Figure 4, adapted from Pessiglione et al., 2006), participants were presented with pairs of stimuli to choose from to maximize their payoffs while undergoing 7 Tesla (7T) MRI. Each stimulus was associated with an 80/20 probabilistic contingency and there were three types of trials: gain, loss and neutral. Participants underwent 90 intermixed trials, with 30 of each trial type. All participants received the same compensation.

### Behavioral analyses

To assess for group differences in learning, participant choices across each trial type (gain/loss) were entered into a linear mixed effects model given by: correctchoice~group×trialtype+trialnumber+(1|participant)

### Computational modeling

A Q-learning algorithm was used to calculate expected value as a function of a state, action pair as given by:

(Equation 1) $${\text{Q}}_{\text{t}}(\text{s},\text{a})={\text{Q}}_{\text{t}-1}(\text{s},\text{a})+\alpha \left(\text{Reward}-{\text{Q}}_{\text{t}-1}(\text{s},\text{a})\right)$$

At the beginning of each trial, Q-values were initiated with a value of zero. Then, these values were updated on each trial based on the difference between the reward received (actual outcome) and the previous value (expected outcome). The difference between the actual outcome and the expected outcome makes up the reward prediction error term. It is scaled by a learning rate (α) which is a free parameter bound between 0–1 that represents the degree to which a participant updates their Q-values. The probability for participants choosing a stimulus is estimated using a standard soft-max rule (Equation 2) where beta (β) serves as a free parameter and controls the level of stochasticity in participants’ choices.


(Equation 2) $$p_{t}^{a}=\frac{\text{exp}\left(\beta Q_{t}^{a}\right)}{\Sigma_{t=1}^{A}\text{exp}\left(\beta Q_{t}^{a}\right)}\{\beta =(0-\infty )\}$$

To test the hypothesis that there are differences in learning from rewards versus losses, three computational models based on Q-learning were developed (see Supplementary Methods).

Model 1: Operates using a single learning to test the hypothesis that individuals update using a single learning rate, regardless of the context they are in:

$${\text{Q}}_{\text{t}}(\text{s},\text{a})={\text{Q}}_{\text{t}-1}(\text{s},\text{a})+\alpha \left(\text{Reward}-{\text{Q}}_{\text{t}-1}(\text{s},\text{a})\right)$$

Model 2: Operates using separate learning rates for gain trials and loss trials to test the hypothesis that differences in learning the value of an action depends on the context, and that individuals update their expectations at different rates based on the context they are in.


$${\text{Q}}_{\text{t}}(\text{s},\text{a})={\text{Q}}_{\text{t}-1}(\text{s},\text{a})+\alpha_{\text{G}}\left(\text{Reward}-{\text{Q}}_{\text{t}-1}(\text{s},\text{a})\right)\;{\text{Q}}_{\text{t}}(\text{s},\text{a})={\text{Q}}_{\text{t}-1}(\text{s},\text{a})+\alpha_{\text{L}}\left(\text{Reward}-{\text{Q}}_{\text{t}-1}(\text{s},\text{a})\right)$$

Model 3: Operates similarly to model 2, using separate learning rates for gain and loss trials. However, this model was specified with 2 separate group-level hyperparameters in order to generate posterior distributions for HC and participants with MDD. Then, depending on the group a participant was in, subject-level parameters were sampled.

### MRI data acquisition and processing

Structural MRI and functional MRI (fMRI) data were acquired using the ultra-high field 7T MRI scanner (Magnetom, Siemens) at the BioMedical Engineering and Imaging Institute, ISMMS, New York. T1-weighted anatomical scans were collected using a dual-inversion magnetization prepared gradient echo (MP2RAGE) sequence^71^ with the following parameters: repetition time (TR)=4500ms, time to echo (TE)=3.37ms. Resting state (i.e. “task-free”) and task-based functional scans were collected during multi-band multi-echo fMRI with the following parameters: TR=2100ms, TE=14.0, 37.87, 61.74ms, 69 near axial slices, 1.5mm isotropic resolution.

After converting all dicoms to nifti files, preprocessing, decomposition and denoising of the functional data were performed using the Multi-Echo Independent Components Analysis (ME-ICA) pipeline^72,73^ (https://bitbucket.org/prantikk/me-ica). By acquiring multiple echoes, ME-ICA leverages the distinct echo time (TE) dependence of the blood oxygen level-dependent (BOLD) signal and various noise sources to enhance signal fidelity. Due to T2* attenuation, the intensity of the BOLD signal will weaken as the TE increases. Thus, multi-echo acquisition allows characterization of the T2* decay curve, which helps distinguish neural activity-related signals from physiological or motion-related artifacts.

Structural T1 images were skull stripped and normalized to the Montreal Neurological Institute (MNI) template space^74^ using the ‘auto_tlrc’ function in AFNI^75^. After ME-ICA denoising, resting state data were coregistered to the structural T1 with ‘align_epi_anat’ function in AFNI, while task-based data were co-registered using either ‘align_epi_anat’ or ‘SSwarper’ functions in AFNI. Finally, resting state data were spatially smoothed with a 3 mm Full Width at Half Maximum (FWHM) Gaussian kernel, and task-based data were smoothed with a 5 mm FWHM Gaussian kernel and scaled.

### Connectivity analysis

Functional connectivity analysis using resting-state fMRI was conducted with a focus on the VTA and seven regions that comprise the mesocorticolimbic network (vmPFC, dmPFC, sgACC, plACC, NAc, BLA, and hippocampus) using Pearson’s correlation, calculated with AFNI’s 3dfim+ and 3dmaskave functions (VTA-to-ROI). Similarly, and in line with previous works^76,77^, task-based functional connectivity analysis was conducted to control for inter-individual variability that may occur during an unconstrained state^77–80^ (i.e. rest) and to amplify the detection of more reliable brain signals with ME-ICA as compared to resting-state data alone.

To assess for group differences in functional connectivity between the VTA and mesocorticolimbic regions in MDD and HC during task-free brain states, VTA-to-ROI connectivity estimates were entered into a group comparison analysis, adjusting for age, sex, medication and preprocessing variability (i.e. daw parameter). Multiple comparisons were corrected using the False Discovery Rate (FDR) approach^81^.

Complementary whole-brain VTA-to-voxel functional connectivity analyses were conducted to assess the voxelwise VTA-to-whole-brain functional connectivity map across all participants and to explore group differences between MDD and HC (Supplementary Figure 1). Whole-brain correction threshold was calculated at voxelwise p<0.005, with a two-sided NN1 threshold of 73 for the analysis of autocorrelation function (ACF) differences (p<0.05).

To examine the relationship between VTA circuitry connectivity within the mesocorticolimbic network and dimensional measures of anhedonia, we performed partial correlation analyses between VTA-to-ROI connectivity and anticipatory (TEPS-A) or consummatory (TEPS-C) anhedonia scores. These analyses controlled for demographic and methodological covariates, including age, sex, medication status, and preprocessing variability. A supplementary confirmatory model included group (MDD vs. HC) as an additional covariate. Then, to examine associations between VTA circuitry functional connectivity and depressive symptoms (MADRS) and anxiety symptoms (STICSA), we conducted partial correlation analyses while controlling for demographic and methodological covariates and group. Separate models were constructed for the functional connectivity between the VTA and each of the seven selected regions of the mesocorticolimbic network in relation to clinical measures. Across all models, corrections for multiple comparisons were applied using the FDR method for a total of seven comparisons.

To assess the relationship between VTA functional connectivity across task-free and task-based brain states, VTA-to-ROI connectivity estimates were subjected to Spearman correlation analysis. Separate spearman correlation analyses were conducted to assess the relationship between VTA-to-ROI connectivity estimates during task-based fMRI and RPE encoding.

### Hormones

To assess the relationship between peripheral gonadal hormone levels and VTA circuitry functional connectivity within the mesocorticolimbic network we performed separate partial correlation analyses relating VTA-to-ROI connectivity estimates to testosterone and estradiol, while controlling for age, sex, medication status, and preprocessing variability, with a second confirmatory model including group as a covariate.

### Dependency Network Analysis (${\text{D}}_{\text{EP}}\text{NA}$)

To assess directional dependencies and interactive influences between the VTA and regions within the mesocorticolimbic network in MDD and across varying levels of anhedonia, we conducted ${\text{D}}_{\text{EP}}\text{NA}$ on resting-state fMRI time series data. ${\text{D}}_{\text{EP}}\text{NA}$ is a graph theory-based network method that models the direction in which variables (i.e. nodes) impact one another by constructing a directed functional graph^82,83^. In our study, ${\text{D}}_{\text{EP}}\text{NA}$ takes as input the time series data from the VTA, amygdala, plACC, sgACC, dmPFC, hippocampus, NAc, and vmPFC and illustrates the connections between the VTA and other regions using arrows to indicate the direction of influence (i.e. whether the VTA influences other regions or is influenced by them). Here, our primary objective was to quantify the overall influence of the VTA on other nodes, as well as the reciprocal influences of these nodes on the VTA, treating the VTA as the central node within the network.

In this approach, each participant’s resting-state functional MRI time series data was extracted from selected nodes within the mesocorticolimbic network. First, the time series of all nodes (i.e. brain regions) were concatenated in sequence into a matrix. Pearson correlation matrices using the timeseries data were then calculated for each participant to evaluate pairwise correlations between each pair of nodes. To reduce the effect of autocorrelation, the diagonal elements of the correlation matrix were adjusted by first replacing values of one with 0.99 and then resetting them to one. Then, we calculated partial correlation coefficients (Equation 3, Figure 1B) between each pair of nodes, vmPFC and NAc to control the influence of a third node, VTA. This allowed us to estimate the direct correlation strength between each pair of nodes. Fisher Z transformation was applied to both Pearson and partial correlations for normalization.

(Equation 3) $$PC(\text{vmPFC},\text{NAc}|\text{VTA})=\frac{C(\text{vmPFC},\text{NAc})-C(\text{vmPFC},\text{VTA})C(\text{NAc},\text{VTA})}{\sqrt{\left[1-C^{2}(\text{vmPFC},\text{VTA})\right]\left[1-C^{2}(\text{NAc},\text{VTA})\right]}}$$

To evaluate the VTA’s centrality within the mesocorticolimbic network we calculated its correlation influence (d) (Equation 4, Figure 1B), computed as the difference between Fisher’s z-transformed full and partial correlation coefficients. Correlational influence reflects the unique contribution of a connection between two nodes after removing the influence of a third node. A larger correlation influence indicates that the observed correlation between two nodes is largely explained by the third node. Any negative values of correlation influence were set to zero.

(Equation 4) d(vmPFC,NAc∣VTA)=C(vmPFC,NAc)-PC(vmPFC,NAc∣VTA)

By averaging the correlation influence of each node on all other node pairs, we constructed a node by node dependency matrix for each participant to capture inter-node dependencies. Here, we specifically focused on the average correlation influence (Equation 5, Figure 1C) of the VTA across all node pairs. For instance, in Figure 1C, we calculated the average correlation influence (D) of the VTA on the vmPFC and its connections with other nodes (R) in the network:

(Equation 5) $$D(\text{vmPFC},\text{VTA})=\frac{1}{n-1}\sum_{R\neq \text{VTA}}^{n-1}d(\text{vmPFC},R|\text{VTA})$$

Specifically, we quantified the influencing degree (Equation 6, Figure 1D) and influenced degree (Equation 7, Figure 1D) of the VTA within the mesocorticolimbic network. The influencing degree of the VTA refers to the total influence exerted by the VTA on other regions, and is determined by the sum of the VTA’s average correlation influence on all other nodes (N) in the network. The higher the influencing degree, the greater its impact on all other regions within the network. Conversely, the influenced degree of the VTA, captures how the VTA was affected by other regions, and defined as the sum of the average correlation influences on the VTA by all other nodes. The higher the influenced degree of a region, the more it was affected by all other regions within the network.

(Equation 6) $$\text{Influencing}\;\text{degree}(\text{VTA})=\sum_{N\neq \text{VTA}}^{n-1}D(N,\text{VTA})$$

(Equation 7) $$\text{Influenced}\;\text{degree}(\text{VTA})=\sum_{N\neq \text{VTA}}^{n-1}D(\text{VTA},\text{N})$$

To ensure robustness, VTA influencing and influenced degrees that deviated more than three standard deviations from the mean were excluded from the analysis (one subject excluded for influencing degree, two subjects excluded for influenced degree). Multivariate linear regression analyses were conducted to compare VTA influencing and influenced degrees between the MDD and HC groups, as well as across different levels of anhedonia, while controlling for age, sex, medication status, and preprocessing variability. Since the analysis focused solely on the VTA node, multiple comparison correction was not required.

To construct directed graphs that reflect the connections and directional dependencies between the VTA and other nodes within the mesocorticolimbic network, comparing across groups and varying levels of anticipatory or consummatory anhedonia, we extracted VTA-to-node pairs from the average correlation influence values in the dependency matrix. We then conducted multivariate linear regression to assess for group differences and variations across anhedonia levels in average correlation influence, controlling for age, sex, medication, and preprocessing variability. Significant VTA-to-node connections ($p_{\text{uncorr}}<0.05$) were represented as edges, and multiple comparison correction was applied (see full results in Supplementary Table 1). Although no connections survived FDR correction except three connections across different levels of consummatory anhedonia (NAc to VTA: $\beta =-0.003,p_{\text{uncorr}}=0.003,p_{\text{FDR}}=0.019;$ dmPFC to VTA: $\beta =-0.003,p_{\text{uncorr}}=0.004,p_{\text{FDR}}=0.019$; amygdala to VTA: $\beta =-0.002,p_{\text{uncorr}}=0.004,p_{\text{FDR}}=0.019$), we still constructed directed graphs centered on the VTA based on the uncorrected results to better visualize the broader network patterns.

#### Task-based first-level imaging analysis

Three sets of first level analyses were conducted using AFNI’s ‘3dDeconvolve’ function. The first general linear model (${\text{GLM}}_{\text{full-RPE}}$) assessed where RPEs are encoded throughout the brain regardless of trial valence and are thus referred to as ‘Full RPE’ (gain, loss and neutral). This model included 3 separate regressors for the onset of stimulus presentation type (gain, loss, and neutral) and 1 regressor for the onset of feedback information across gain, loss, and neutral trials, which was parametrically modulated by the RPEs generated from our best-fitting computational model in a model-based fMRI approach^84^. The onset of button press was also modeled to capture any variance associated with a motor response. A second, exploratory GLM (${\text{GLM}}_{\text{gain-loss-RPE}}$) assessed where brain representations of gain/loss trial RPEs are separately encoded. This model included 3 separate regressors for the onset of stimulus presentation type (gain, loss, and neutral), 2 regressors for the onset of feedback information parametrically modulated by RPEs during gain and loss trials, and 1 regressor for the onset of feedback information during neutral trials. Similarly to ${\text{GLM}}_{\text{full-RPE}}$, the onset of button press was modeled to capture any variance associated with a motor response.

In a hypothesis driven approach, a region of interest (ROI) analysis was conducted with the VTA, NAc and vmPFC and BLA selected as a priori ROIs. Average beta weights for feedback-related regressors with parametric modulation were extracted from the mask for each ROI. First, unpaired, two sample t-tests, or Wilcoxon signed-rank tests when data were not normally distributed, were run for each ROI across ${\text{GLM}}_{\text{full-RPE}}$ and were FDR corrected for multiple comparisons. Then, the relationship between each ROI and anhedonia/anxiety as measured by the TEPS/STICSA was explored using Pearson or Spearman correlation, where appropriate.

#### Task-based second-level imaging analysis

${\text{GLM}}_{\text{full-RPE}}$ and ${\text{GLM}}_{\text{gain-loss-RPE}}$ were explored at the second level for whole-brain group analysis of the feedback-related regressors with parametric modulation only. The whole brain correction threshold was calculated at voxelwise p < 0.001 and cluster > 109 voxels for alpha < 0.05 using AFNI’s autocorrelation function for mitigating against spatial autocorrelation and false positives^85^. Using the same approach for ${\text{GLM}}_{\text{gain-loss-RPE}}$ the whole brain correction threshold was calculated at voxelwise p < 0.001 and cluster > 112 voxels for alpha < 0.05.

## Supplementary Files

This is a list of supplementary files associated with this preprint. Click to download.

- BeltranLiSupplementaryMaterials.docx

## Figures and Tables

**Table 1.** Demographics and clinical features.

	HC (N=41) mean/count (SD/%/range)	MDD (N=42) mean/count (SD/%/range)
Age (years)	30.73 (8.54)	28.31 (6.75)
Sex (female)	23 (56.1%)	24 (57.1%)
Age of onset (years)	--	15.9* (5 – 40)
Duration of current depressive episode (months)	--	55.74* (1 – 336)
Comorbid anxiety disorder	--	30 (71.4%)
Medicated	--	20 (47.62%)
MADRS	.63* (0 – 4)	27.9* (10 – 41)

On average, participants with MDD were characterized by moderate depression based on the Montgomery-Asberg Depression Rating Scale (MADRS). *SD* = standard deviation, *HC* = *healthy controls, MDD = major depressive disorder*

* N=38 HC, N=41 MDD

## Data Availability

The data that support the findings of the current study are available from the corresponding author upon reasonable request.

## Code Availability

The underlying code for this study is not publicly available but may be made available to qualified researchers on reasonable request from the corresponding author.
